# Transcriptome analysis reveals multiple targets of erythritol-related transcription factor EUF1 in unconventional yeast *Yarrowia Lipolytica*

**DOI:** 10.1186/s12934-024-02354-9

**Published:** 2024-03-12

**Authors:** DA. Rzechonek, M. Szczepańczyk, I. Borodina, C. Neuvéglise, AM. Mirończuk

**Affiliations:** 1 Laboratory for Biosustainability, Institute of Environmental Biology, Wrocław University of Environmental and Life Sciences, Wrocław, Poland; 2 Department of Life Sciences (LIFE), Chalmers University of Technology, Göteborg, Sweden; 3 The Novo Nordisk Foundation Center for Biosustainability, Technical University of Denmark, Kgs. Lyngby, Denmark; 4 INRAE, Institut Agro, SPO, University Montpellier, Montpellier, France

**Keywords:** *Yarrowia Lipolytica*, Erythritol, RNA-Seq, Leucine degradation, Glyoxylate cycle, Erythrose reductase

## Abstract

**Background:**

Erythritol is a four-carbon polyol with an unclear role in metabolism of some unconventional yeasts. Its production has been linked to the osmotic stress response, but the mechanism of stress protection remains unclear. Additionally, erythritol can be used as a carbon source. In the yeast *Yarrowia lipolytica*, its assimilation is activated by the transcription factor Euf1. The study investigates whether this factor can link erythritol to other processes in the cell.

**Results:**

The research was performed on two closely related strains of *Y. lipolytica*: MK1 and K1, where strain K1 has no functional Euf1. Cultures were carried out in erythritol-containing and erythritol-free media. Transcriptome analysis revealed the effect of Euf1 on the regulation of more than 150 genes. Some of these could be easily connected with different aspects of erythritol assimilation, such as: utilization pathway, a new potential isoform of transketolase, or polyol transporters. However, many of the upregulated genes have never been linked to metabolism of erythritol. The most prominent examples are the degradation pathway of branched-chain amino acids and the glyoxylate cycle. The high transcription of genes affected by Euf1 is still dependent on the erythritol concentration in the medium. Moreover, almost all up-regulated genes have an ATGCA motif in the promoter sequence.

**Conclusions:**

These findings may be particularly relevant given the increasing use of erythritol-induced promoters in genetic engineering of *Y. lipolytica.* Moreover, use of this yeast in biotechnological processes often takes place under osmotic stress conditions. Erythritol might be produce as a by-product, thus better understanding of its influence on cell metabolism could facilitate processes optimization.

**Supplementary Information:**

The online version contains supplementary material available at 10.1186/s12934-024-02354-9.

## Background

Erythritol is a four-carbon polyol produced by a number of yeasts. It is commercially used as a sweetener, so the improvement of its biotechnological production has been the subject of many research [1–3] Synthesis proceeds using enzymes of the non-oxidative phase of pentose-phosphate pathway, while the final step is catalyzed by erythrose reductases [4]. *Yarrowia lipolytica* is an unconventional, oleaginous yeast, increasingly used in the food industry, and has a potential to serve as a model organism for metabolism of polyols and lipids [5]. The importance of erythritol for survival of this yeast might be indicated by the presence of multiple homologs of erythrose reductase. Deletion of eight of them results in 91% decrease in erythritol synthesis, but so far, it has not been possible to obtain a strain completely unable to produce erythritol [6].

The best known role of erythritol is protection against osmotic stress. HOG signaling pathway induces synthesis of polyols in presence of salts or high concentration of carbon sources [7]. Impaired erythritol production, caused by the damage to HOG or the aforementioned deletions of numerous erythrose reductases, resulted in significantly increased osmo-sensitivity. However, such strains were able to grow under osmotic stress conditions if erythritol was supplemented in the media [6, 8].

Erythritol can also serve as a carbon source, used after depletion of glycerol or glucose. Assimilation is possible thanks to four enzymes of the erythritol utilization pathway: Eyd1, EyI1, Eyk1 and EyI2 [9–11]. Their expression is regulated by the transcription factor Euf1 (Erythritol Utilization Factor) and genes encoding all five proteins are group together in a cluster [10]. Damage or deletion of *EUF1* gene could significantly impair or completely stop the ability to grow on erythritol – depending on culture conditions [12]. So far enzymes of utilization pathway were the only known targets of Euf1, however there are indications that it may also play a role in erythritol-based osmoprotection [8].

Erythritol is not a common carbon source, but could be used by a group of other unconventional yeasts. Utilization enzymes have been described in oleaginous *Lipomyces starkeyi* [13], and BLAST analyses indicate the presence of similar proteins in several other genera, including *Galactomyces*, *Rhodotorula* and *Puccinia*. Moreover, the study of erythritol metabolism in yeast could potentially provide a model for better understanding its function in higher organisms. Erythritol is used as a sweetener, and for a long time it was thought to be harmless and almost not metabolized in the human body. However, more recent studies indicate that its presence in the blood is an early marker of weight gain [14] and may even contribute to severe cardiovascular complications [15].

The aim of the study is to test whether Euf1 regulates more genes than previously identified. We presume that the acquired information will contribute to a better understanding of erythritol metabolism and its role in *Y. lipolytica*, but also other eukaryotic organisms.

The study is based on a transcriptome comparison of two strains of *Y. lipolytica*: MK1 and K1, that differ by a spontaneous mutation. Strain K1 lacks functional Euf1, because the premature stop codon was introduced by the point mutation – only 200 out of 951 amino acids could be translated [12]. Using the K1 strain instead of performing a deletion of the entire *EUF1* allowed to avoid the introduction of additional modifications to the genes used as markers. The most common marker systems in *Y. lipolytica* are based on damage to the uracil (*URA3*) or leucine (*LEU2*) synthesis pathways [16]. Genes of these pathways could have been potential targets of Euf1; therefore we did not want to interfere with them.

## Methods

### Yeast strains and media

Strains used in the study are the very closely related *Y. lipolytica* K1 [17] and *Y. lipolytica* MK1 [18], obtained from the Department of Biotechnology and Food Microbiology at Wrocław University of Environmental and Life Sciences. In strain K1 there is a point mutation, creating a stop codon at the beginning of the gene encoding Euf1 [12].

YPD liquid medium, consisting of 10 g/L yeast extract (Merck, Germany), 20 g/L glucose (Chempur, Poland), 20 g/L peptone (Merck, Germany), was used for inoculum preparation and storage. YPD strain cultures with 25% glycerol addition were stored at -80 °C. YNB without amino acids (Sigma-Aldrich, Germany) was a base for most of the liquid media used in the study and it was used in a concentration of 6.8 g/L. Carbon sources added to the YNB media were: glycerol (Wratislavia-Biodiesel), glucose (Chempur, Poland) or erythritol (Młyn Oliwski, Poland).

### Shake-flask cultures

Shake-flask experiments were performed in 0.3 L flasks with baffles containing 0.05 L of medium kept on a rotary shaker at 240 rpm, 28 °C. Two types of media were used for these experiments: YNB + 50 g/L glycerol + 50 g/L erythritol or YNB + 50 g/L erythritol + 50 g/L glucose. 3 g/L CaCO_3_ was also added in order to prevent a drop in pH during culture. The inoculum was grown for 72 h in YPD medium or 24 h in YNB medium + 50 g/L glycerol (28 °C, 200 rpm). It was diluted to starting OD_600_ approximately 2. The cultures were performed in triplicate. Samples were collected every 24 h.

### Bioreactor cultures

The inoculum was prepared in two steps. First strains were grown in 10 mL tubes containing 5 mL of YPD medium (24 h, 28 °C, 200 rpm) and later in a 300 mL flask containing 100 mL of YNB + 50 g/L glycerol (24 h, 28 °C, 200 rpm). 100 mL of inoculum was added to 900 mL of medium in the bioreactor up to a final working volume of 1 L and starting OD_600_ approximately 2 (which is around 1.2 × 10^7^ cells/mL). Media used in experiments were YNB + 50 g/L glycerol + 50 g/L glucose or YNB + 50 g/L glycerol + 50 g/L erythritol. The cultures were performed in triplicate. The batch bioreactor cultures were performed in 5 L Biostat B Plus fermenters (Sartorius, Germany) with the working volume of 1 L, at temperature 28 °C, stirring ratio 800 rpm and aeration 1 L/min. pH was maintained at 5.6 by the automatic addition of 20% NaOH.

Samples were harvested every 12 h and additionally half an hour after depletion of the first carbon source (glycerol) in the medium. The moment of changing the carbon source was easily determined by observing the amount of NaOH solution automatically added to the bioreactor. In case of a switch to glucose, NaOH uptake decreased noticeably, while after switch to erythritol the uptake was completely stopped. The timing of the moment of glycerol depletion was further confirmed by HPLC analysis.

### Analytical methods

10 mL samples, collected from the bioreactor to determine biomass content, were centrifuged at 5500 *g*. The pellet was washed with distilled water, harvested by filtration and dried at 105 °C. The concentrations of glucose, polyols and citric acid in the supernatant from the samples were determined by HPLC using a HyperRez Carbohydrate H + Column (Thermo Scientific, Waltham, MA) coupled to a UV (λ = 210 nm) (Dionex, Sunnyvale, USA) and a refractive index detector (Shodex, Ogimachi, Japan).

### RNA extraction and sequencing

Samples for RNA isolation were collected from bioreactors after 24 h of culture and after depletion of the first carbon source in the medium (32–36 h of culture). 10 mL of sample was collected in a 50 mL centrifuge tube and centrifuged for 1 min at 4 °C. The supernatant was discarded, the pellet was immediately frozen in liquid nitrogen and stored at – 80 °C. Cells were disrupted with glass beads in Percellys24 homogenizer. RNA was extracted using an RNase kit (Qiagen, Germany) according to the manufacturer’s protocol. Unfortunately, due to technical issues some of the samples were taken with a longer delay – about an hour after the switch.

Library preparation and sequencing were performed by the NGS lab at the Novo Nordisk Foundation Center for Biosustainability. Sequencing was carried out using the NextSeq 500 system (Illumina) in pairend mode and 75 bp length of reads.

### Transcriptome data analysis

The RNA-seq data were processed using KBase [19]. The quality of reads was checked with FastQC v 0.11.5 (Bioinformatics Group at the Babraham Institute). Adapter and low quality sequences were removed by Trimmomatic v0.36 [20]. Reads from the same samples were merged by Merge Reads Libraries v1.0.1 and annotated to the reference genome using HISAT v2.1.0 [21]. The reference genome was *Y. lipolytica* CLIB122 (GenBank assembly accession: GCA_000002525.1). Subsequently StringTie v2.1.5 [22] was used to assemble the transcripts, determine levels of expression and provide normalized expression matrix (TPM and FPKM). The comparison between samples from different conditions and principal component analysis were made by DESeq2 v1.20.0 [23]. The *p*-value ≤ 0.01 and log2 (fold change) ≥ 1.5 cutoffs were applied to select the genes of interest.

After the principal component analyses, it was observed that the four samples taken with a delay clearly differ from the other measurements from their series. Thus it was decided to remove them and repeat the transcriptomic analyses from the start. Consequently, some of them were performed on two instead of three biological replicates. These conditions were: K1/glycerol-glucose/24 h, K1/glycerol-glucose/33 h, K1/glycerol-erythritol/33 h, MK1/glycerol-erythritol/33 h.

Probable functions and GO terms of genes were assigned based on GRYC database, InterPro [24] and Panther v14.0 [25]. The pathways were constructed with the KEGG pathway database [26]. Heatmaps were prepared using the Heatmapper [27] online tool. Promoter sequences from the whole *Yarrowia* clade were collected from the GRYC database by courtesy of Cecile Neuvéglise and Hugo Devillers. Conservative motifs in the promoter sequences were searched with YEASTRACT + [28].

## Results

### Optimization of culture conditions

The first step of the study was to determine the optimal culture conditions and sampling time for RNA isolation. The effect of *EUF1* damage on erythritol utilization varies greatly depending on culture conditions. It is likely that the assimilation is also affected by other factors that have not been described yet. To rule out their potential influence, we looked for culture conditions in which strain K1 (without functional Euf1) was completely unable to utilize erythritol. This approach required usage of media with additional carbon source to obtain enough biomass of K1 for RNA isolation.

**Fig. 1:**
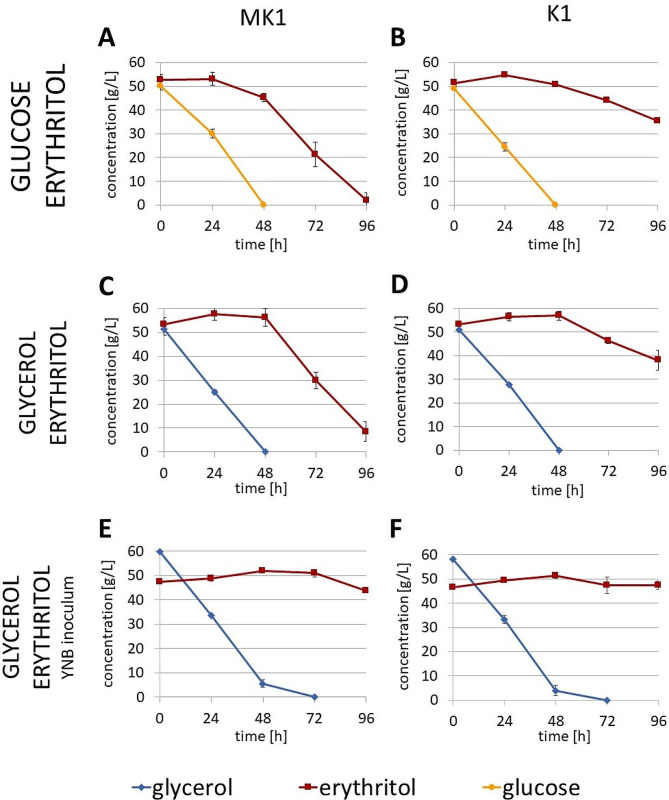
Carbon source consumption by *Y. lipolytica* strains MK1 and K1 during shake flask cultures. Yeast nitrogen base was used in each medium. (**A**) MK1, glucose/erythritol, YPD inoculum, (**B**) K1, glucose/erythritol, YPD inoculum, (**C**) MK1, glycerol/erythritol, YPD inoculum, (**D**) K1, glycerol/erythritol, YPD inoculum, (**E**) MK1, glycerol/erythritol, YNB/glycerol inoculum, (**F**) K1, glycerol/erythritol, YNB/glycerol inoculum. Experiments were performed in triplicate. Error bars indicate standard deviation

Initially, two combinations of YNB-based media were tested: glucose/erythritol and glycerol/erythritol. The cultures were carried out in baffle flasks (Fig. 1), since this has a positive impact on erythritol synthesis and utilization [4]. In the culture on glucose/erythritol medium, glucose was used first and the rate of utilization did not differ between MK1 (Fig. 1A) and K1 strains (Fig. 1B). During this time, the amount of erythritol fluctuated slightly. After glucose depletion at 48 h, strain MK1 began rapid erythritol utilization. Strain K1 also assimilated erythritol, but at a much slower rate – when the culture was terminated after 96 h, there was still 35.3 ± 0.5 g/L.

Erythritol was also utilized second when cultures were performed in glycerol/erythritol medium (Fig. 1C-D). During the consumption of glycerol, the concentration of erythritol in the medium slightly increased. This could be explained by the production of erythritol by *Y. lipolytica* as a stress response, because 50 g/L of glycerol is enough to induce osmotic stress [8]. Only after the complete depletion of glycerol did erythritol uptake begin – fast in case of MK1 and slower for K1. Thus, it was possible to pinpoint the exact moment of the switch to erythritol utilization. For this reason, the glycerol/erythritol combination was chosen for the study.

Another issue was to further decrease the utilization of erythritol by the K1 strain, so various culture parameters were tested. The inoculum used for starting the culture proved to be crucial. The change of the inoculum medium from YPD to YNB with 5% glycerol slowed down the subsequent consumption of erythritol. As much as 43.8 ± 0.9 h/L was still present in the medium, when the culture of the MK1 strain was stopped at 96 h (Fig. 1E). In the case of strain K1 no erythritol utilization was observed (Fig. 1F). The YNB inoculum was therefore used in further bioreactor cultures.

**Fig. 2:**
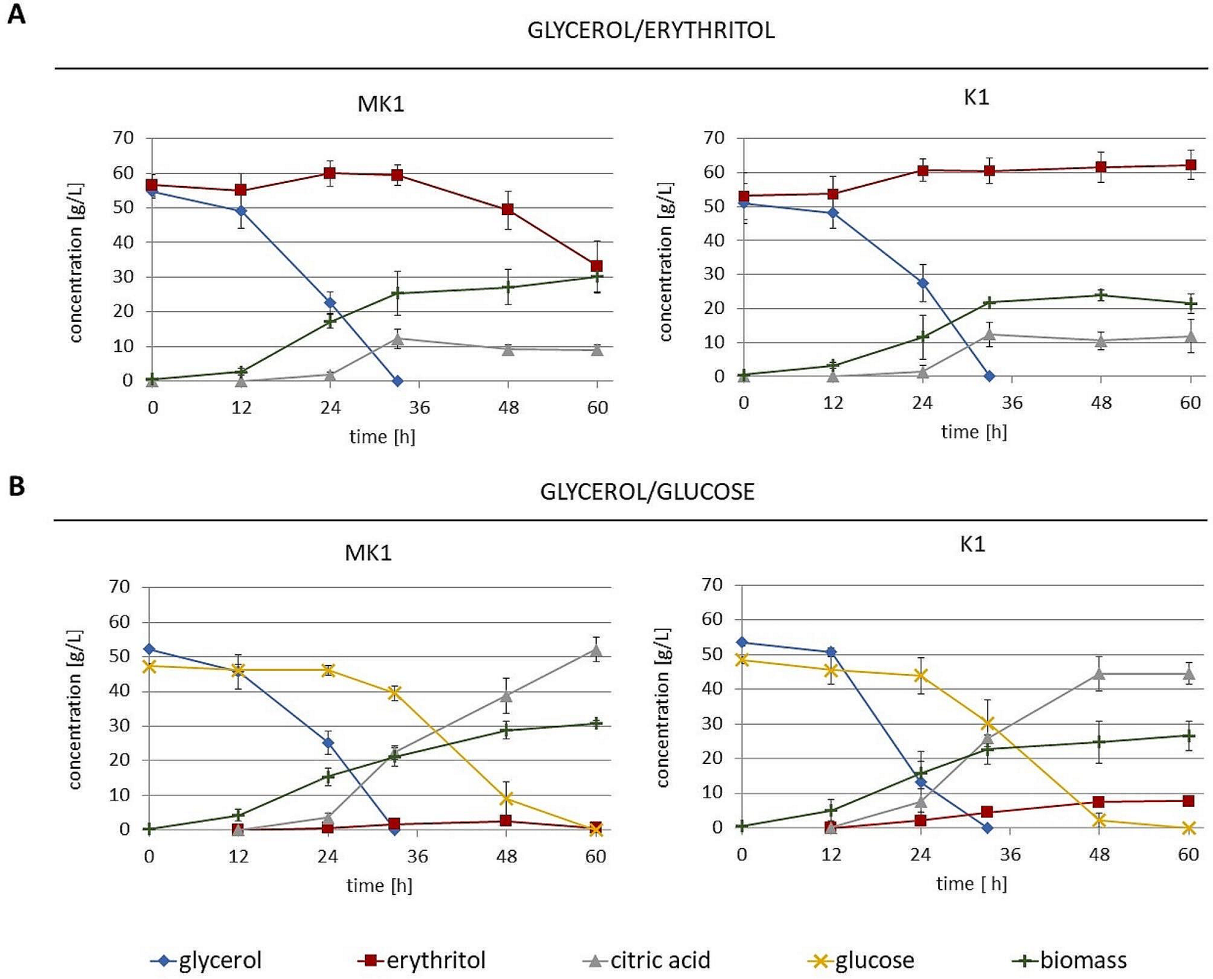
Bioreactor cultures of *Y. lipolytica* strains MK1 and K1. YNB without amino acids was used in each medium. (**A**) glycerol and erythritol used as a carbon source, (**B**) glycerol and glucose used as a carbon source. Experiments were performed in triplicate. Error bars indicate the standard deviation

The goal of the bioreactor cultures was to obtain biomass for RNA isolation. Constant control of parameters like pH, oxygenation and automatic addition of NaOH (to prevent acidification) allowed to precisely identify the moment of carbon source switch. In contrast to shake-flask cultures, significant amount of citric acid was produced during the growth on glycerol. Acid production stopped when all the glycerol was used after about 33 h (Fig. 2A). Then MK1 strain began slow utilization of erythritol, while K1 was not able to use this carbon source. Two time points were chosen to collect biomass for RNA isolations: 24th hour of culture, and 30 min after glycerol depletion.

There was also a question if Euf1 could influence transcription when erythritol was not present in the environment in high concentrations. To test this, we performed bioreactor cultures with YNB medium containing glycerol/glucose (Fig. 2B). Glycerol was again utilized first, but the uptake of glucose started before glycerol was completely consumed. A compound produced in significant amount was citric acid. Its concentration increased during both glycerol and glucose consumption, however in different rates. The uptake of NaOH solution slowed down after depletion of glycerol (data not shown). The differences between MK1 and K1 strains were not as distinct as in the previous experiment, but still apparent. Erythritol was not initially present in the medium, but was produced in the course of culture. When samples for RNA isolation were collected after the depletion of glycerol, erythritol concentrations reached 1.7 ± 0.6 g/L for MK1 strain and 4.5 ± 0.8 g/L for K1. Its final concentration for the K1 strain was 7.7 ± 0.8 g/L, while MK1 reassimilated erythritol before the termination of the culture. Moreover, the K1 strain began glucose assimilation earlier.

### Differential expression

**Fig. 3:**
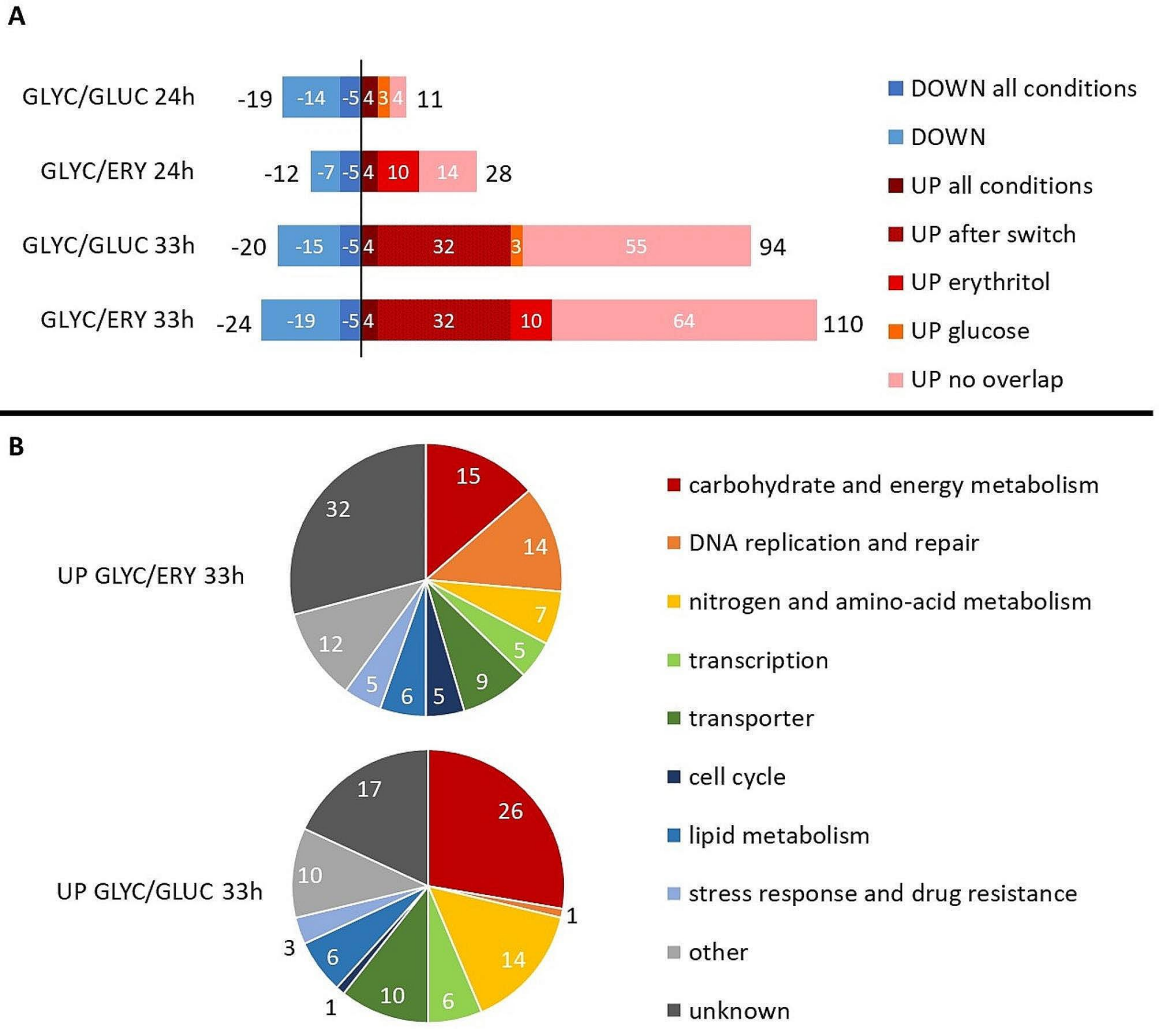
(**A**) Up- and down-regulated genes with information of the number of genes that overlap in different conditions. (**B**) Putative functional groups of genes up-regulated genes at 33 h. Upregulated genes have higher expression in strain MK1 compared to K1

RNA sequences were analyzed using the KBase platform [19]. The first step was to determine which genes undergo the greatest changes in the transcriptional level. Samples from each culture condition were compared between MK1 and K1 strains. Higher expression in the MK1 strain is further defined as up-regulation. Statistics on the number of genes whose transcriptional changes exceeded the threshold (*p*-value ≤ 0.01 and log2 (fold change) ≥ 1.5) are shown in Fig. 3A, while a full list of these genes with their putative functions and GO terms can be found in Supplementary File 1.

The number of down-regulated genes was similar in all conditions, ranging from 12 to 24 (Fig. 3A). Five of the genes were down-regulated in all tested conditions, with four of them grouped in a cluster (*YALI0E05819g* – putative RNA helicase, *YALI0E05841g –* putative membrane traffic protein, *YALI0E05863g* – putative mitochondrial genome maintenance protein, *YALI0E05907g –* putative peroxisomal membrane protein). Much larger differences were observed in the case of up-regulation. In samples taken at 24 h, there were 11 genes for GLYC/GLUC medium and 28 for GLYC/ERY. In contrast, when the carbon source was switched at 33 h, the values increased to 110 up-regulated genes for erythritol medium and 94 for glucose.

Only 4 genes were up-regulated in all tested conditions. These were: *YALI0A09262g* (encoding an alkaline peptidase precursor), *YALI0C12364g* (Nrg1 transcriptional repressor), *YALI0E31757g* (transcription factor) and *YALI0C17567g* (unknown function). Up-regulation of the *YALIC12364g* gene is a likely cause of downregulation of cluster of the genes mentioned earlier. Experiments supporting this hypothesis are presented in Supplementary Figures - Fig S2.

Thirty-two genes were up-regulated at 33 h in both erythritol and glucose media. Based on information collected in either NCBI, GRYC, KEGG or Panther databases, as many as 28 of them were assigned a probable function (Supplementary File 1). This group includes the entire erythritol utilization cluster, potential transporters (7 genes), and some enzymes of amino acid degradation pathways and other transcription factors (3 genes).

Up-regulation of many genes was observed in only one of the tested conditions, especially after depletion of glycerol. To better illustrate these differences, genes overexpressed at 33 h were classified into categories based on their likely role in metabolism (Fig. 3B, Supplementary File 1). On erythritol medium, as many as 32 genes could not be assigned any probable function, much more than on glucose (17 genes). In addition, groups overexpressed on erythritol but almost absent on glucose are related to DNA replication and repair (14 genes) and cell cycle (5 genes).

On glucose, two biggest groups of overexpressed genes are associated with carbohydrate and energy metabolism (26 genes) and nitrogen and amino acid metabolism (14 genes). However, an interesting feature of many of the genes was that their transcription on erythritol was actually much higher. The probable activation of transcription by a high concentration of erythritol obliterated the differences between MK1 and K1 strains on erythritol medium, which, although noticeable, turned out to be below the threshold. Therefore, only on glucose medium it was possible to observe clear up-regulation by Euf1. Examples of this will be discussed further for specific genes.

In the following sections, the results will be presented mainly in the form of heatmaps that were prepared on the basis of FPKM (fragments per kilobase million) values, that have been further normalized within rows. These allow simultaneous presentation of trends in numerous genes, but they do not represent the differences in transcription levels between these genes, so the average FPKM values were also added to the heatmaps. A table with FPKM values of all genes can be found in Supplement File 2.

### Erythritol metabolism

**Fig. 4:**
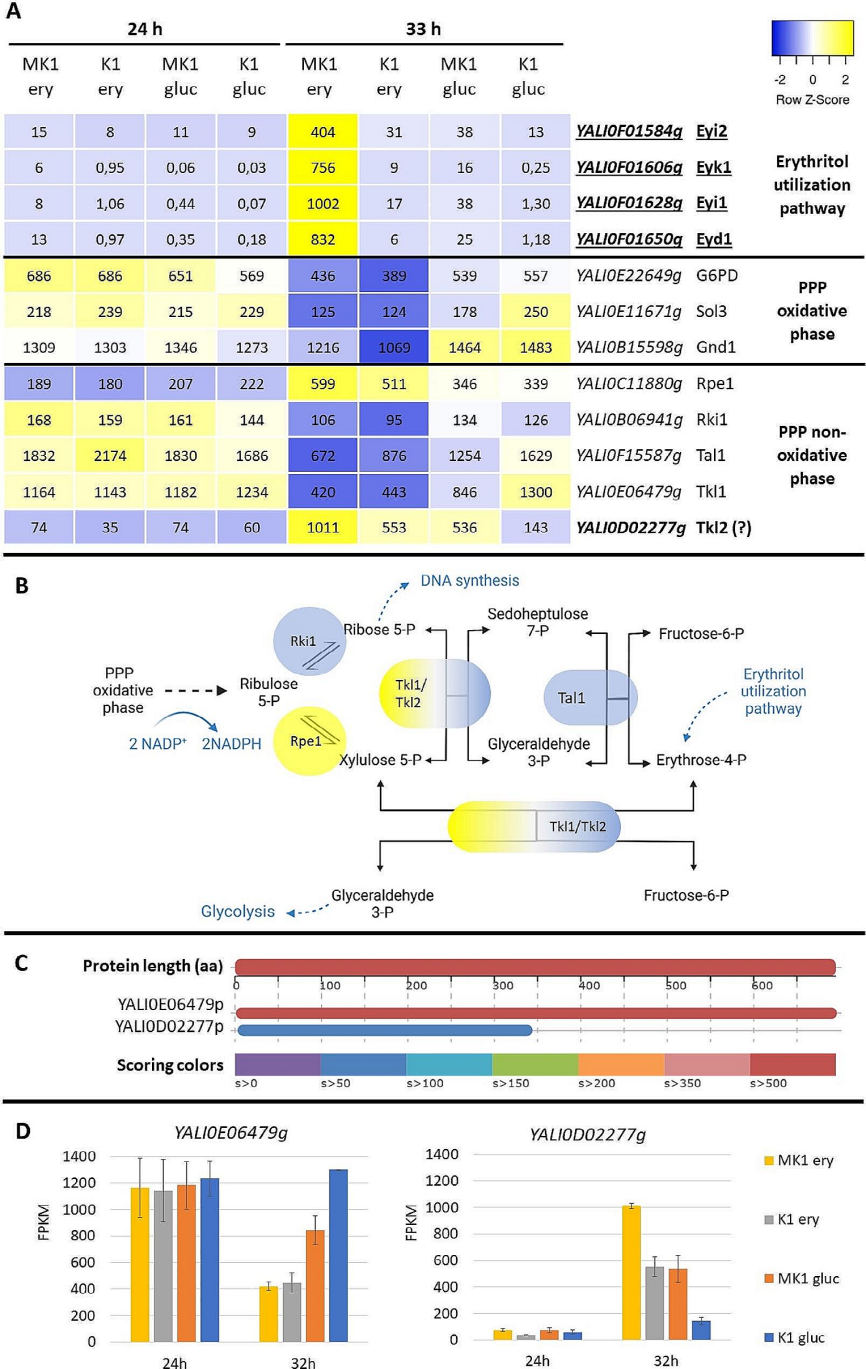
Investigating the role of the pentose phosphate pathway in erythritol utilization: (**A**) expression of genes from erythritol utilization pathway and PPP presented in the form of a heatmap, (**B**) scheme of PPP non-oxidative phase, (**C**) graphic summary of BLASTP of transketolase protein YALI0E06479b in *Y. lipolytica* E150 revealing another putative transketolase variant, YALI0D2277b (from GRYC), (**D**) expression of *YALI0E06479g* and *YALI0D02277g*. Font modifications in the name of the genes indicate that they were significantly up-regulated at 33 h: underline – on erythritol medium, bold – on glucose medium. Genes involvement in PPP pathway was based on KEGG Pathways

The first known Euf1-regulated genes encoded enzymes of the erythritol utilization pathway: *EYI1*, *EYI2*, *EYK1* and *EYD1* [10]. All four genes were up-regulated at 33 h on both erythritol and glucose medium. However, despite the clear up-regulation by Euf1, FPKM values on glucose medium for strain MK1 remained low – not exceeding 40 for any of the genes (Fig. 4A). Only the presence of a high erythritol concentration in the medium allowed for a sharp increase in expression– exceeding 400 FPKM for all four genes and reaching 1000 ± 460 for *EYI1*. This indicates that both Euf1 and a high concentration of erythritol are required for induction of the utilization pathway expression. Moreover, a comparison of the expression for strain MK1 between 24 and 33 h (Fig. 4A) indicates the existence of an additional control: even when erythritol was present in high concentrations, the expression of the whole pathway remained low when glycerol was still present in the media.

In earlier studies, it was proposed that enzymes of the utilization pathway convert erythritol to erythrose-4-P [29], which is then incorporated into cell metabolism via the pentose-phosphate pathway (PPP). Therefore, we subjected the PPP genes to targeted transcriptional analysis. As it turned out, the transcription of all enzymes of the PPP oxidative phase (*G6PD, SOL3, GND1*) decreased after the switch to erythritol at 33 h (Fig. 4A).

The omission of the oxidative phase was not unexpected, since erythrose-4-P is an intermediate of the non-oxidative phase (Fig. 4B). However, most of the initially tested PPP non-oxidative genes (D-ribose-5-phosphate isomerase – *RKI1*, transaldolase – *TAL1*, transketolase – TKL1) also showed reduced transcription after depletion of glycerol, the only exception being *RPE1 -* ribulose-phosphate 3-epimerase gene (Fig. 4A). *RKI1*, *TAL1* and *TKL1* also had lower expression on erythritol compared to glucose.

However, there was one other gene that could be involved in the PPP pathway. *YALI0D02277g* was up-regulated by Euf1 and displayed similarity to the transketolase gene *YALI0E06479g* (Fig. 4C). The resulting protein was half as large, but contained a thiamin diphosphate-binding fold domain, crucial for transketolase activity. A comparison of the transcription levels of the *YALI0E06479g* and *YALI0D02277g* genes indicates that they might complement each other (Fig. 4D). The transcription of *YALI0E06479g* was high under most of the tested conditions, but when it decreased during growth on erythritol, there was an increase in *YALI0D02277g* transcripts. In addition, these changes appear to be dependent on both the presence of high concentrations of erythritol in the medium and the Euf1 factor.

Another interesting enzyme from the PPP pathway is ribulose-phosphate 3-epimerase – Rpe1. The changes in expression of its gene are contrary to the rest of the pathway – it increases after glycerol depletion and is slightly higher on erythritol. Euf1 does not seem to affect *RPE1* in any way, but this enzyme may still be very important for erythritol utilization. It catalyzes the formation of xylulose-5-P, which together with erythrose-4-P is one of the substrates for transketolase. Thus, Rpe1 and Tkl2 together could enable the processing of most of erythroso-4-P and bypassing the remaining enzymes of the PPP pathway (Fig. 4B).

**Fig. 5:**
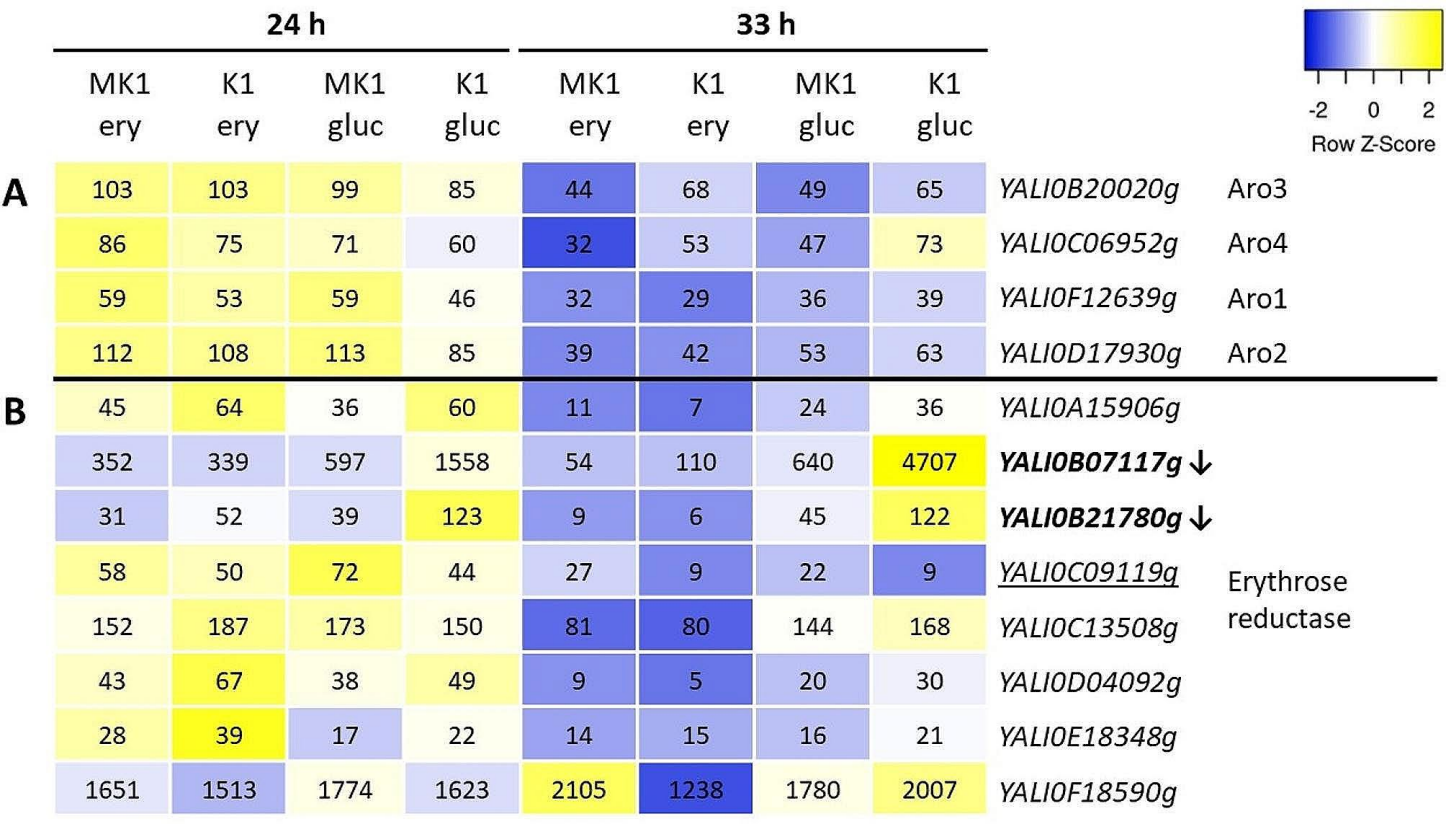
(**A**) Expression of genes involved in Shikimate pathway. (**B**) Expression of genes encoding erythrose reductase, with average FPKM values. Font modifications in the name of the genes indicate significant changes of expression: underline – on erythritol medium, bold – on glucose medium. Arrows indicate, that genes are down-regulated in presence of Euf1

Erythrose-4-P can also be used as a precursor for the synthesis of aromatic amino acids via the shikimate pathway (Larroude et al. 2021). In order to test whether Euf1 can influence the redirection of carbon flow toward this pathway, we checked the transcript levels of the first four enzymes of the shikimate pathway (*YALI0B20020g –* Aro3, *YALI0C06952g –* Aro4, *YALI0F12639g –* Aro1, *YALI0D17930g –* Aro2). No significant differences in expression were observed for any of them, neither between the MK1 and K1 strains, nor between the erythritol and glucose media (Fig. 5A). This indicates that Euf1 is not involved in all aspects of processing erythritol derivatives.

Finally it was examined if Euf1 could affect erythritol synthesis. We checked transcription levels of eight genes encoding erythritol reductases (Fig. 5B). Most of them, showed higher expression at 24 h, that lowered after switch to the second carbon source, especially erythritol. However, this group of genes was not completely homogeneous.

The presence of Euf1 clearly affected the expression of *YALIB07117g* and *YALI0B21780g*, which were down-regulated on glycerol/glucose medium. The absence of Euf1 resulted in a particularly high rise in the expression of *YALIB07117g*, which in strain K1 reached as much as 4700 ± 900 FPKM. On the other hand, *YALI0C09119g* was up-regulated on erythritol medium. This result was surprising, but the overall expression of this gene was low, compared to the other reductases. However, the most distinct was the expression pattern of the *YALI0F18590g*, which remained at a very high and stable level under all tested conditions - not significantly affected by Euf1, time of sampling or the type of substrate.

### The unexpected targets of Euf1

**Fig. 6:**
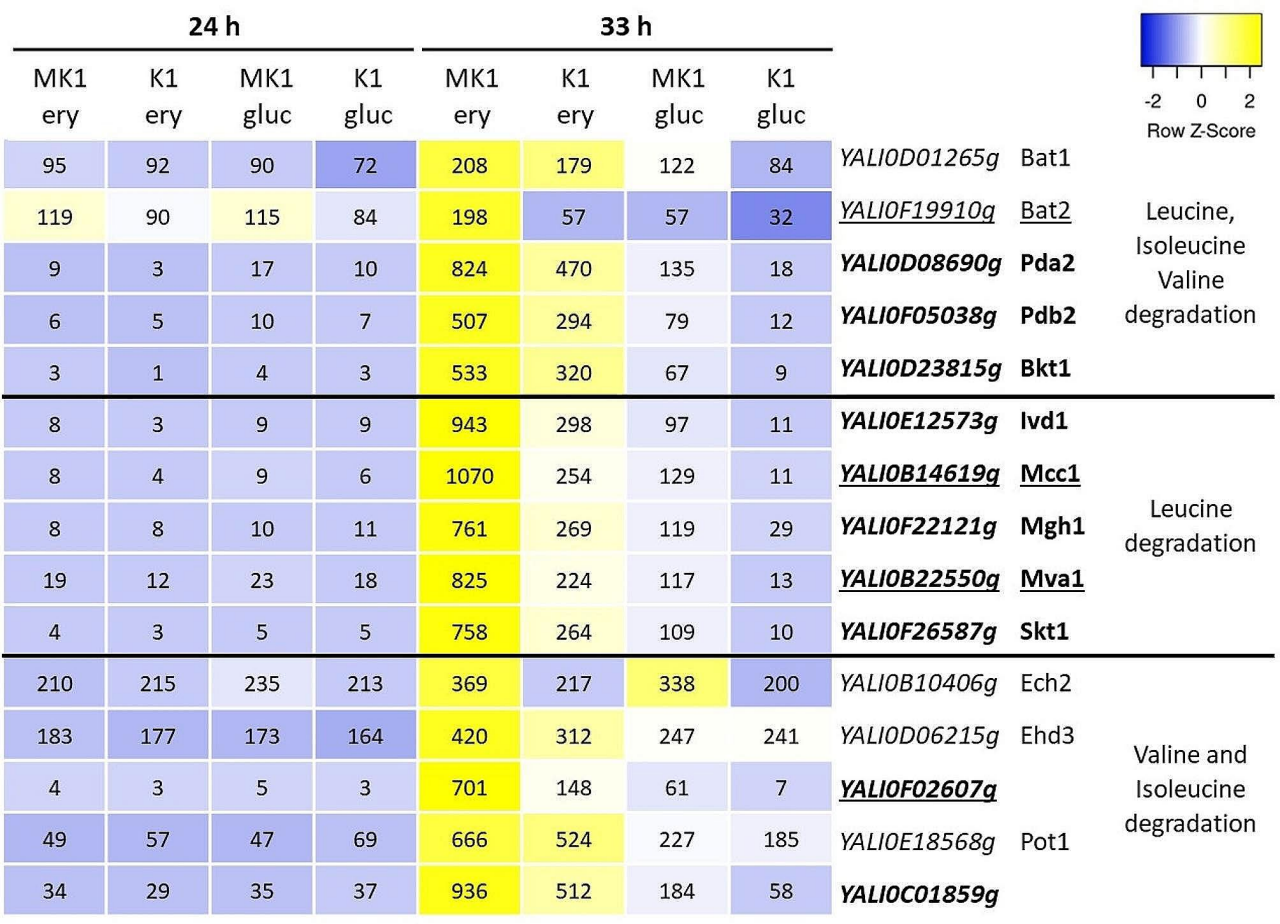
Expression of genes from leucine, isoleucine and valine utilization pathways presented in the form of a heatmap, with the average FPKM values for each sample. Font modifications in the name of the genes indicate that they were significantly up-regulated at 33 h: underline – on erythritol medium, bold – on glucose medium. Genes involvement in branch-chain amino acids degradation pathways was based on KEGG Pathways

Euf1 affects the expression of many genes that are difficult to directly link to erythritol utilization. The most prominent example is the up-regulation of the entire degradation pathway of branched-chain amino acids: leucine, isoleucine and valine (Fig. 6). The initial degradation steps of all three amino acids are conducted by the same enzymes (encoded by the genes *YALI0D01265g*, *YALI0F19910g*, *YALI0D08690g* and *YALI0D23815g*). All of them are up-regulated in strain MK1, and comparison between glucose and erythritol media indicates that expression is higher on erythritol. Of this group, only *YALI0F19910g* stands out, having a fairly high level of expression during growth on glycerol at 24 h. The leucine utilization enzymes (encoded by *YALI0E12373g*, *YALI0B14619g*, *YALI0F22121g*, *YALI0B22550g*, *YALI0F26587g*) show an extremely similar expression pattern – which is highly dependent on both EUF1 and the presence of erythritol. In the K1 strain on glucose, the FPKM values of all these genes do not exceed 12, while in MK1 on erythritol they reach 800–1000 FPKM. Of all the enzymes involved in leucine degradation, only BAT1 (*YALI0D01265g*) did not exceed the 1.5-(log2)fold change threshold on any of the substrates.

Euf1 also has some effect on valine (*YALI0B10406g*, *YALI0D06215g*, *YALI0F02607g*) and isoleucine degradation (*YALI0B10406g*, *YALI0E18568g*, *YALI0C1859g*), but it is weaker. Significant up-regulation was observed only for *YALI0F02607g* and *YALI0C01859g*.

**Fig. 7:**
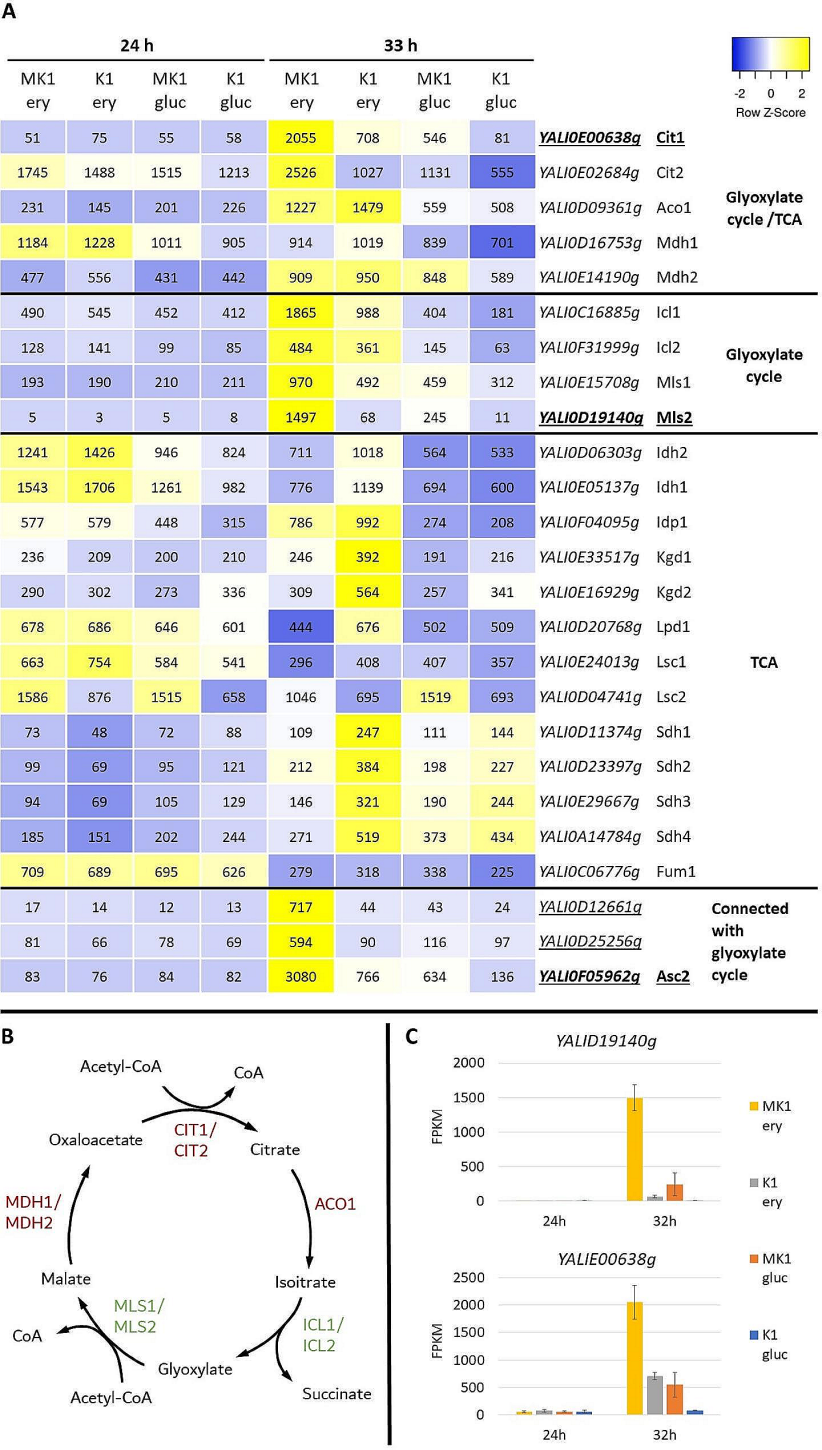
Investigating the impact of EUF1 and erythritol on glyoxylate cycle and TCA cycle: (**A**) Expression of genes presented in the form of a heatmap and average FPKM values for each sample, (**B**) scheme of glyoxylate cycle, green color indicates enzymes characteristic for glyoxylate cycle, red color indicates enzymes that appear also in TCA cycle, (**C**) expression of *YALI0D19140g* and *YALI0E00638g.* Genes involvement in TCA and glyoxylate cycles was based on KEGG Pathways

Another unexpected pathway up-regulated by Euf1 or high concentrations of erythritol is the glyoxylate cycle (Fig. 7A). This cycle allows the utilization of bicarbonate residues formed from the conversion of acetate, or the degradation of fatty acids. It is sometimes referred to as a variant of the tricarboxylic acid cycle, and both cycles involve the enzymes citrate synthase, aconitase and malate dehydrogenase (Fig. 7B).

Two genes encoding putative enzymes involved in the glyoxylate pathway are significantly up-regulated: *YALI0E00638g* (2-methyl citrate synthase – Cit1) and *YALI0D19140g* (malate synthase – Mls2). Expression of *YALI0D19140g* is almost entirely dependent on the presence of Euf1 (Fig. 7C): at 24 h it does not exceed 10 FPKM, while it increases rapidly after the switch to erythritol (up to 1497 ± 189,4573 FPKM), but only for strain MK1. Other genes of the glyoxylate cycle also exhibit differences in expression between MK1 and K1 strains or between glucose and erythritol media (Fig. 7A first panel), with the only exception being *YALI0D16753g* (mitochondrial malate dehydrogenase – Mdh1). The different expression of *MDH1* can be explained by mitochondrial localization of its protein, which indicates that it participates in TCA, but not in the glyoxylate cycle. This contrasts with Mdh2, which is localized in the peroxisome or cytoplasm, depending on the splicing [30].

Transcript levels of enzymes present only in the TCA were also examined (Fig. 7A second panel) and provided a distinct contrast to the glyoxylate cycle. Among the TCA enzymes, strain K1 has slightly higher expression, although the differences were not sufficient to be considered as down-regulation by Euf1.

Up-regulation of putative enzymes providing precursors to the glyoxylate pathway was also noted: glycolate oxidase (*YALI0D12661g*), glyoxylate reductase (*YALI0D25256g*) and acetyl-CoA synthetase (*YALI0F05962g*) (Fig. 7A third panel). The leucine degradation pathway also terminates at acetyl-CoA, which can be incorporated into the glyoxylate cycle.

Here arises the question of how (if at all) the branched-chain amino acid degradation or glyoxylate cycle could be linked to erythritol utilization. Moreover, there are a few other genes whose increased expression is surprising and difficult to explain (Supplementary file 1), such as putative formate dehydrogenases (*YALI0A12353g*, *YALI0B19976g*, *YALI0B22506g*, *YALI0C08074g*, *YALI0C14344g*, *YALI0E15840g*, *YALI0F13937g*), alcohol dehydrogenases (*YALI0A16379g*, *YALI0E17787g*) and aldehyde dehydrogenase (*YALI0E00264g*). The difficulty in proposing possible connections lies in the fact that the putative function of some of these enzymes has been proposed solely on the basis of similarity to better characterized proteins from other yeast, while their actual role may be different.

### Transporters

The last group we would like to investigate are the transporters. Fifteen potential transporters were up-regulated after switches to glucose or erythritol. Among them were putative polyamine transporters (*YALI0A15576g* and *YALI0B21142g*), a siderophore iron transporter (*YALI0D05401*g) and a mitochondrial organic acid transporter (*YALI0E34672g*). However, we were most interested in trying to identify proteins that may have been involved in the transmembrane transport of erythritol. We focused on six proteins containing MFS (major facilitator superfamily) domains.

**Fig. 8:**
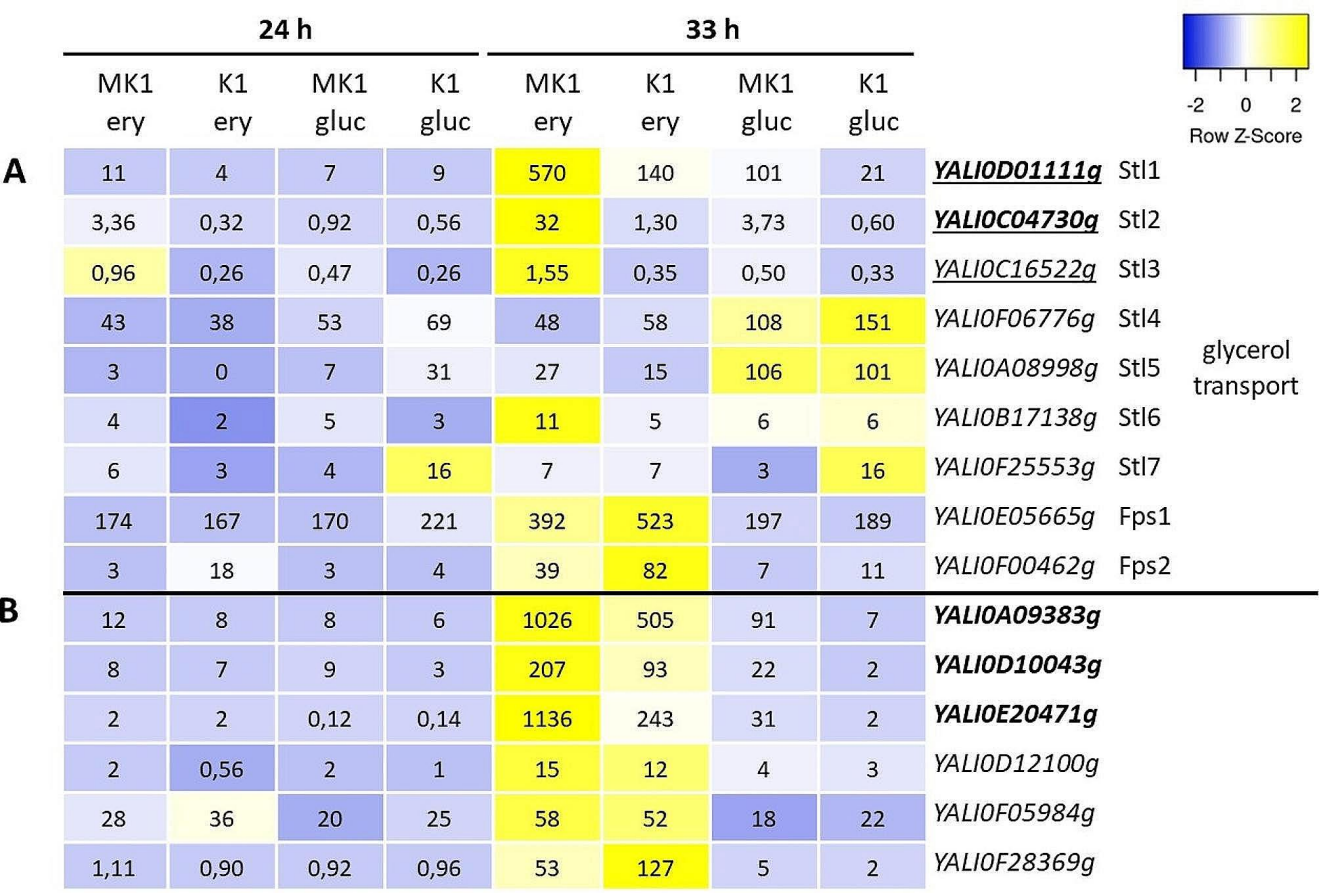
Transporters and aquaporins investigated in regard to erythritol transport: (**A**) genes probably involved in transport of glycerol, (**B**) homologous protein on unknown functions. Font modifications in the name of the genes indicate that they were significantly up-regulated at 33 h: underline – on erythritol medium, bold – on glucose medium

Three of them have already been under investigation as potential glycerol transporters, due to certain homology to the Stl1 protein [31]: *YALI0C04370g*, *YALI0D01111g* and *YALI0C16522g* (Fig. 8A). These three genes have very different levels of expression – for strain MK1 after switching to erythritol it is 570 ± 32 FPKM for *YALI0D01111g*, 32 ± 8 FPKM for *YALI0C04370g* and only 1.6 ± 0.3 FPKM for *YALI0C16522g*. Given the structural similarity of erythritol and glycerol, it cannot be ruled out that both polyols may be transported by the same proteins; however, it should be noted these genes had very low expression at 24 h, when glycerol was used as a carbon source. In order to investigate it further, we decided to check other proteins potentially involved in glycerol transport: more homologs of Stl1 (*YALI0F06776g*, *YALI0A08998g*, *YALI0B17138g*, *YALI0F25553g*) and aquaporins Fps1 (*YALI0E05665g*) and FPS2 (*YALI0F00462g*). The other Stl1 homologs not only showed no significant changes in expression associated with the presence of Euf1, but had lower expression on erythritol compared to glucose (Fig. 7A). Expression of aquaporins was also not dependent on Euf1, but it was higher on erythritol medium.

Other genes that raised our interest were *YALI0A09383g*, *YALI0D10043g* and *YALI0E20471g* (Fig. 8B). Their expression was so strongly activated by high concentrations of erythritol that the up-regulation by Euf1 was noted only on glucose medium. However, the combination of both Euf1 and erythritol in the medium led to the highest expression – for *YALI0A09383g* it was 1025 ± 436 FPKM for the MK1 strain on erythritol.

These three genes shared some homology and were just a part of a larger group of dozens of similar putative transporters. Examination of the remaining homologs showed that a further three of them (*YALI0D12100g*, *YALI0F05984g and YALI0F28369g*) have higher expression on erythritol medium compared to glucose, although Euf1 does not affect them. According to the GRYC database, they show a very weak similarity to proteins characterized in *S. cerevisiae* or *Neurospora crassa*, responsible for transporting molecules such as allenoate (*YALI0A09383g*), tartrate (*YALI0D10043g*, *YALI0E20471g*), and nicotinic acid (*YALI0D12100g*, *YALI0F05984g* and *YALI0F28369g*). Even if they are not related to the transport of erythritol itself, this group might be worth further investigation.

### Common motifs in promoter regions

The final step of the study was to test whether up-regulated genes share conserved motifs within their promoters that could be potential Euf1 binding sites. For this purpose, we looked for promoter regions that were evolutionarily conserved in the *Yarrowia* clade (12 species: *Y. alimentaria*, *Y. galli, Y. phangngensis*, *Y. yakushimensis*, *Y. hollandica*, *Y. bubla*, *Y. lipolytica*, *Y. osloensis*, *Y. porcina*, *Y. deformans*, *Y. divulgata*, *Y. keelungensis*).

The search for conserved motifs began with a cluster of erythritol utilization pathway genes. The common promoter region of the *YALI0F01606g* and *YALI0F01628g* (Fig. S2A) genes had already been subjected to similar analyses in a study devoted to the preparation of an erythritol-induced promoter [32]. At that time, two conserved motifs of 11 and 18 bp in length were identified.

Such long motifs were difficult to find in the promoters of other genes, but their shortened and split elements turned out to be much more widespread. We identified three motifs conserved throughout the erythritol utilization cluster: ATGCA (or its reverse TGCAT), CGGAT (reverse ATCCG) and CGGCTT (reverse AAGCCG). However, there were some variations: in promoters of *YAL0F01584g* and its orthologs motif ATGCA occurred in 2–4 repeats. In contrast, it was much less common in the promoter of *YALI0F01650g* and its orthologs. The distribution of these motifs in the promoter regions of the erythritol cluster is presented in Supplementary Figures – Fig S2. Next we checked how common are these motifs in promoter regions of 168 genes up-regulated in *Y. lipolytica* at 33 h. ATGCA was present in 92% of them, CGGAT in 74% and CGGCTT in 33% (Table 1).

Finally the comparisons were made for promoters of the whole *Yarrowia* clad. Not all of the genes had easily identifiable orthologs in all 12 species. We collected promoters of 146 of the 168 genes and in most cases the comparison was performed on 9–12 promoter fragments of 1,000 bp in length. We considered a motif as a conservative, when it was present in promoters of at least 75% of collected orthologs of a given gene and in a similar position to the start-codon.

The ATGCA motif proved to be the most prevalent. For 146 of the checked genes it was present in at least 75% of orthologs, and for 76 of them it was also in the conservative position. Other two motifs were less common – CGGAT was conservative for 23 genes and CGGCTT only for 11 of them (Table 1). More information about presence of motifs in the *Yarrowia* clad can be found in Supplementary file 3.

**Table 1:** Motifs commonly present in promoter regions of genes up-regulated by Euf1

Motif	Present in Y. lipolytica promoter	Conserved in promoters from Yarrowia cluster	Present in majority of promoters from Yarrowia cluster
ATGCA	155 (92%)	76 (52%)	135 (92%)
CGGAT	125 (74%)	23 (16%)	67 (46%)
CGGCTT	55 (33%)	11 (8%)	18 (12%)

## Discussion

The main question regarding the Euf1 factor was whether its function is limited to regulation of erythritol utilization. Performed RNAseq analyses revealed upregulation of a significant number of genes that appeared to have no obvious connection to erythritol metabolism. However, the study was designed so that comparisons could be made not only between MK1 and K1 strains, but also between the used carbon sources – glucose and erythritol. This second approach showed that the activity of Euf1 is closely related to the presence of erythritol in the environment. Up-regulation of 94 genes was observed on glucose medium (in which erythritol was still present in small amounts), but almost all of them reached high transcription levels only on erythritol medium. Thus, it is reasonable to suspect that Euf1 regulates how the presence of erythritol affects other processes in the cell.

However, comparisons between 24 and 33 h of cultures indicate the existence of an additional level of control. During growth on glycerol, the expression of most genes regulated by Euf1 remained very low. Inhibition of the erythritol utilization pathway might be considered as an example of catabolite repression that prevents the simultaneous consumption of several carbon sources [33]. Glucose repression is a well-described feature of *S. cerevisiae*. In *Y. lipolytica*, where glycerol is the more preferred carbon source, glycerol-induced repression has been studied, although the factors responsible for this phenomenon have not yet been firmly identified [34, 35]. Therefore, it is worth considering other potential causes.

The cultures were carried out in batch reactors where all substrates were added only once at the beginning of the culture. This means that between 24 and 33 h nutrient depletion was occurring. Nitrogen availability is a factor that has a huge impact on gene transcription. It was observed that changing the C/N ratio from 20 to 40 caused increased expression of genes encoding Cit2 (*YALI0E02684*g), transporters YALI0C04730p and YALI0D01111p and the entire erythritol utilization pathway [35]. In addition, the increasing amount of biomass in the reactor may have affected the access of oxygen to cells (even though the reactor was continuously oxygenated with a steady stream of air). Disturbances in dissolved oxygen concentration coupled with glycerol limitation may result in increased expression of glyoxylate cycle-related genes (*YALI0E15708g* – Mls1, *YALI0D19140g* – Mls2, *YALI0D12661g*) [36].

Among the most interesting results are changes in the transcription of genes of the pentose-phosphate pathway. PPP can, parallel to glycolysis, process sugars or polyols, but it also has other important roles in cell metabolism. The oxidative phase generates NADPH, while the non-oxidative phase provides precursors for the synthesis of nucleic acids (ribose-5 phosphate) or aromatic amino acids (erythrose-4-phosphate). Thus, reactions can proceed in different directions, depending on the current demand for these components in the cell [37]. The presence of several transketolase isoforms is not unusual among yeast and might be a way of control the balance of the PPP. *S. cerevisiae* has two, one of which is clearly dominant [38]. In *Moniliella megachiliensis*, two isoforms with complementary roles have been described, with changes in expression associated with the response to oxidative stress [39].

In *Y. lipolytica* and some other unconventional yeasts, PPP is also involved in the production of erythritol in response to osmotic stress [40]. It might create a need for additional control over the pathway. Transketolase Tkl1 (*YALI0E06479g*) proved to be important for synthesis of erythritol, as its overexpression caused a significant increase in production [4]. The reactions of the non-oxidative phase of PPP are reversible, so it was expected that Tkl1 would also participate in processing of erythrose-4-P during erythritol utilization. Yet, during growth on erythritol, the expression of Tkl1 and almost all other PPP enzymes decreased.

However, Euf1 induced an increase in the expression of a potential isoform of transketolase, encoded by the gene *YALI0D02277g*. The resulting protein is half the size of Tkl1, so probably cannot completely replace it. We have made multiple, unsuccessful attempts to delete Tkl1 (data not shown), so we suspect that such a modification is lethal. Therefore, YALI0D02277p might play auxiliary role by taking over only part of the function of transketolase when the erythritol utilization pathway provides excessive erythrose-4-P.

Euf1 affected the expression of several other transcription factors. Our attention was particularly drawn to the repressor Nrg1, up-regulated by Euf1 under all tested conditions. Mao et al. studied the effect of this protein on filamentous growth of *Y. lipolytica*. They performed the deletion of the gene and RNA-seq analysis. Nrg1 turn out not to be a key player for the dimorphic switch, but the available transcriptomic data [41] proved to be crucial for the interpretation of our results. Among the genes repressed by Nrg1 there are 5 encoding erythrose reductases: *YALI0B07117g, YALI0B21780, YALI0A15906, YALI0D04092, YALI0E18348*. This explains the observed down-regulation of these genes (Fig. 5B), as well as the higher concentration of erythritol in bioreactors with K1 strain (Fig. 2B). The differences in the amount of erythritol in cultures of MK1 and K1 was initially explained only by the fact that MK1 can utilize it quicker. However this is probably an overlay of two mechanisms. Euf1 not only induces erythritol utilization, but, through up-regulation of Nrg1, can also blocks its simultaneous synthesis.

The most unexpected result was the Euf1-induced upregulation of degradation pathways of branched-chain amino acids, particularly leucine. It is difficult to directly link this pathway to erythritol processing, so we focused on the role of leucine in the cell. In addition to being an essential amino acid, changes in its concentration can contribute to the activation of signaling pathways. In *S. cerevisiae*, leucine activates the TORC1 complex [42], which promotes cell proliferation and protein anabolism [43]. In *S. pombe*, leucine limitation may be one of the triggers of autophagy, which is also associated with TOR [44]. Finally, negative control between lipid accumulation and leucine biosynthesis was observed in *Y. lipolytica* [45]. Thus, leucine deficiency may signal unfavorable conditions for cell proliferation. Such a temporary slowdown in biomass growth was observed after glycerol depletion and a switch to erythritol (Fig. 2A), even in the case of the MK1 strain. This prompts us to consider why the activation of erythritol utilization and mechanisms resulting in potential decreased proliferation would be activated by the same transcription factor.

It should be noted that erythritol is not a carbon source found in significant amounts in nature. In the case of some yeasts, it is produced in response to osmotic or oxidative stress [46–48], thus its presence may signal unfavorable conditions. In addition, erythritol provides some protection against the effects of osmotic stress [8]. So, although suitable as a carbon source, consuming it too quickly could be detrimental to cells. Euf1 up-regulates the enzymes of the glyoxylate cycle (Fig. 6), which may indicate preparation of the cell for parallel use of other carbon sources, such as lipids, or acetate. Orthologs of some genes regulated by Euf1 were also involved in adaptation to diverse stress conditions. In *S. cerevisiae CIT1* and *MLS1* were upregulated in strains adapted to oxidative stress [49], and upregulation of genes encoding alcohol and aldehyde dehydrogenases were observed in strains resistant to 2-Phenylethanol [50].

The information presented in this paper might have practical applications in the genetic engineering of *Y. lipolytica*. In recent years, synthetic promoters induced by erythritol have gained popularity [32, 51]. Induction requires the addition of erythritol, and to ensure that it is not rapidly disposed of, one of the enzymes of the utilization pathway (Eyk1) is also knocked out [51, 52]. This could lead to additional accumulation of erythritol formed naturally during fermentation, which in turn might contribute to gene up-regulation by Euf1. Obviously, synthetic promoters are designed to be more powerful than naturally occurring ones, but unintentional upregulation of pathways such as the glyoxylate cycle or degradation of branched-chain amino acids might disrupt the experiments. This could be particularly relevant when employing strains auxotrophic to leucine, also popular in *Y. lipolytica* genetic engineering [16]. Induced promoters are a very useful tool, so we hope that the present results will facilitate their use at the stages of planning experiments and interpreting results.

## Conclusions

The activity of the transcription factor Euf1 is closely related to the regulation of erythritol’s effect on the cell. It activates the utilization of this polyol, and indirectly inhibits its simultaneous synthesis. Euf interacts with dozens of other genes, but its ability to trigger their high transcription is dependent on the concentration of erythritol in the environment. This may indicate that erythritol produced under osmotic stress can influence numerous cellular processes through Euf1. The occurrence of osmotic stress induced by high substrate concentrations can emerge during biotechnological processes in which *Y. lipolytica* is increasingly used. Moreover, given the increasingly popular trend of introducing erythritol-induced promoters, understanding the effects of this polyol on the cell can be an important tool for process optimization.

### Electronic supplementary material

Below is the link to the electronic supplementary material.

Supplementary Material 1

Supplementary Material 2

Supplementary Material 3

Supplementary Material 4

Supplementary Material 5

## Data Availability

No datasets were generated or analysed during the current study.
