# Supplementary material for: Transcriptome analysis reveals multiple targets of erythritol-related transcription factor EUF1 in unconventional yeast Yarrowia Lipolytica
Source: Microb Cell Fact. 2024 Mar 12;23:77. doi: 10.1186/s12934-024-02354-9 (PMC10935855; doi:10.1186/s12934-024-02354-9)
Supplement: Supplementary file 2 — Supplementary Material 2 [file 12934_2024_2354_MOESM2_ESM.docx]

**Transcriptome analysis reveals multiple targets of erythritol-related transcription factor EUF1 in unconventional yeast *Yarrowia lipolytica***

**Rzechonek DA^a1^, Szczepańczyk M^a^, Borodina I^b^, Neuvéglise C^c^, Mirończuk AM^a*^**

1. Wrocław University of Environmental and Life Sciences, Institute of Environmental Biology, Laboratory for Biosustainability, Wrocław, Poland
2. The Novo Nordisk Foundation Center for Biosustainability, Technical University of Denmark, Kgs. Lyngby, Denmark.
3. INRAE, Institut Agro, SPO, University Montpellier, Montpellier, France
4. Present address: Department of Life Sciences (LIFE), Chalmers University of Technology, Gothenburg, Sweden, [dorota.rzechonek@chalmers.se](mailto:dorota.rzechonek@chalmers.se)

*Corresponding author: [aleksandra.mironczuk@upwr.edu.pl](mailto:aleksandra.mironczuk@upwr.edu.pl)


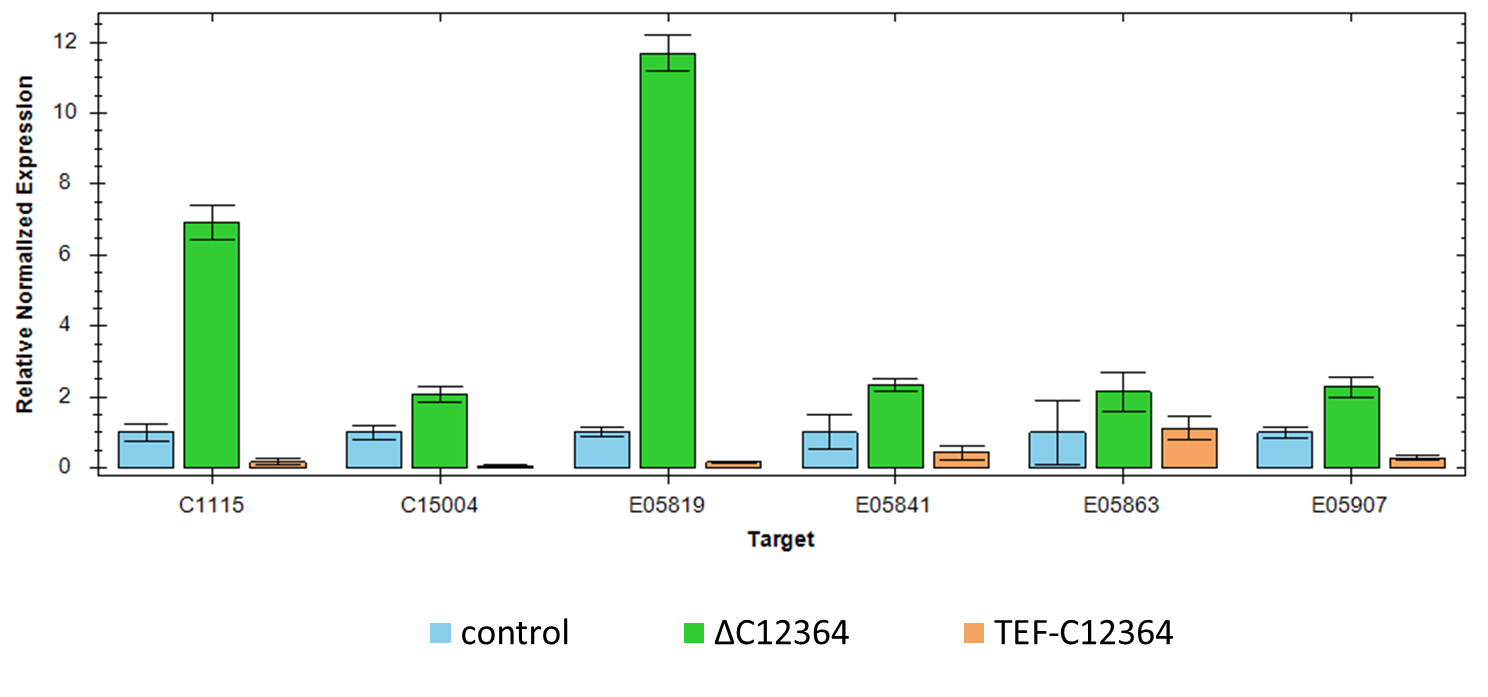


**Fig S1**. Results of qPCR targeting genes *YALI0C11165g, YALIC15004g, YALI0E05819g, YALI0E05841g, YALIE05863g and YALI0E05907g* performed on three *Y. lipolytica* strains AMM+ (control), AMM *C12364∆* (deletion of *YALI0C12364g*) and AMM TEF-C12364 (overexpression of *YALI0C12364g*).

*YALI0C115g, YALIC15004g, YALI0E05819g, YALI0E05841g, YALIE05863g and YALI0E05907g* were all down-reguleted in RNA-seq comparison between strains MK1 and K1. We suspected that the downregulation might be connected with transcriptional repressor encoded by *YALI0C12364g*. The repressor had higher expression in strain MK1, which might lead to downregulation of its targeted genes.

To test this possibility we prepared strains with deletion (AMM *C12364∆*) and overexpression (AMM TEF-C12364) of *YALI0C12364g*. All AMM strains are derivatives of MK1. Their detailed description will be presented in a publication that is currently under preparation.

Strains were grown in shake-flasks on YNB medium with 10% glycerol. Samples for RNA isolation were taken after 24h. The q-PCR results are presented as Relative Normalized Expression in comparison to AMM+ control strain (where *YALI0C12364*g was not modified) . Deletion of *C12364g* repressor resulted in higher expression of the tested genes, while the overexpression of *C12364g* leads to lower expression. It supports the assumption that the downregulation of these genes in MK1 strain is a result of upregulation of *YALI0C12364g* by Euf1.


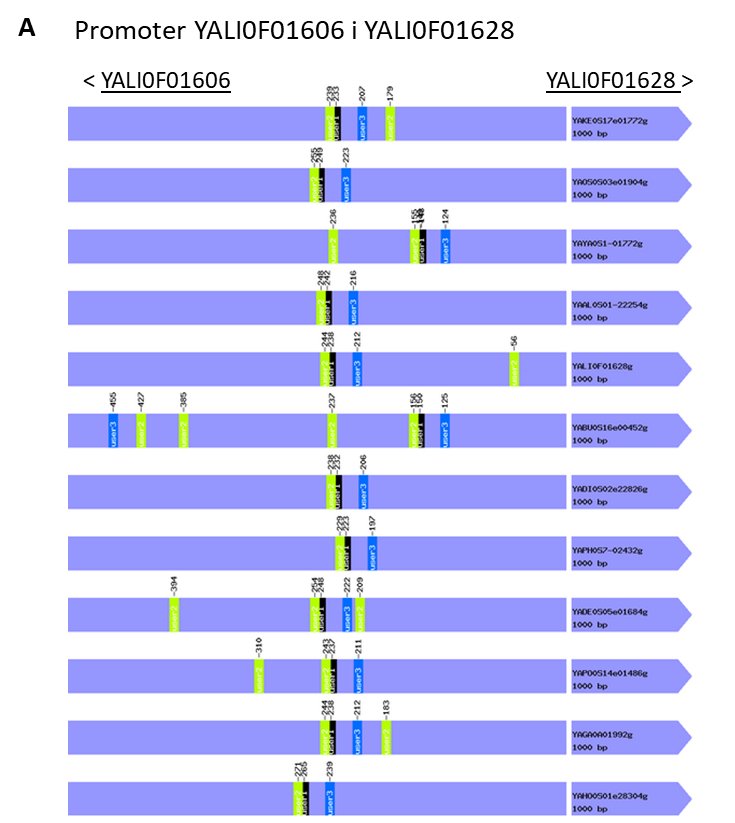

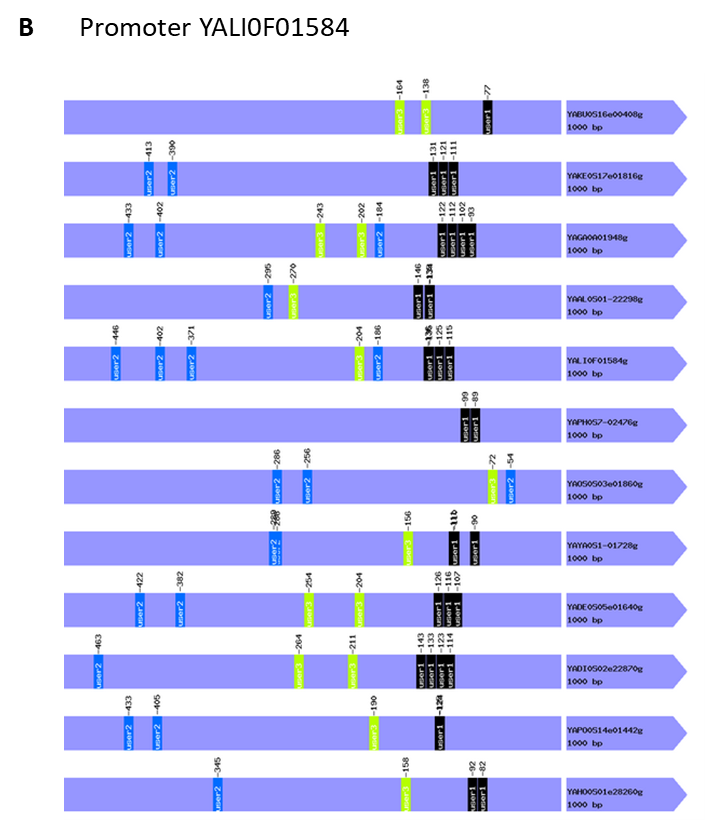

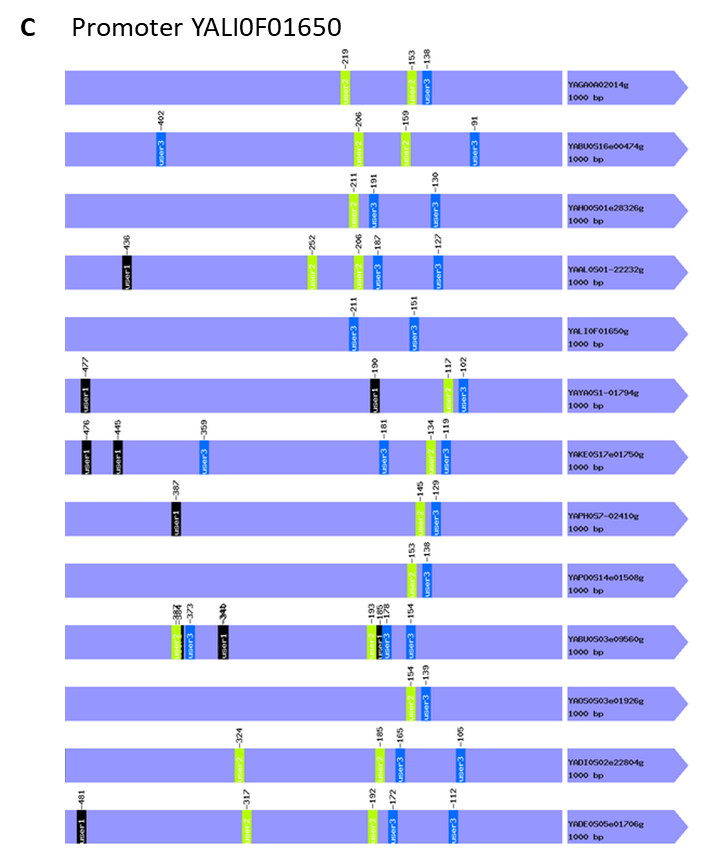

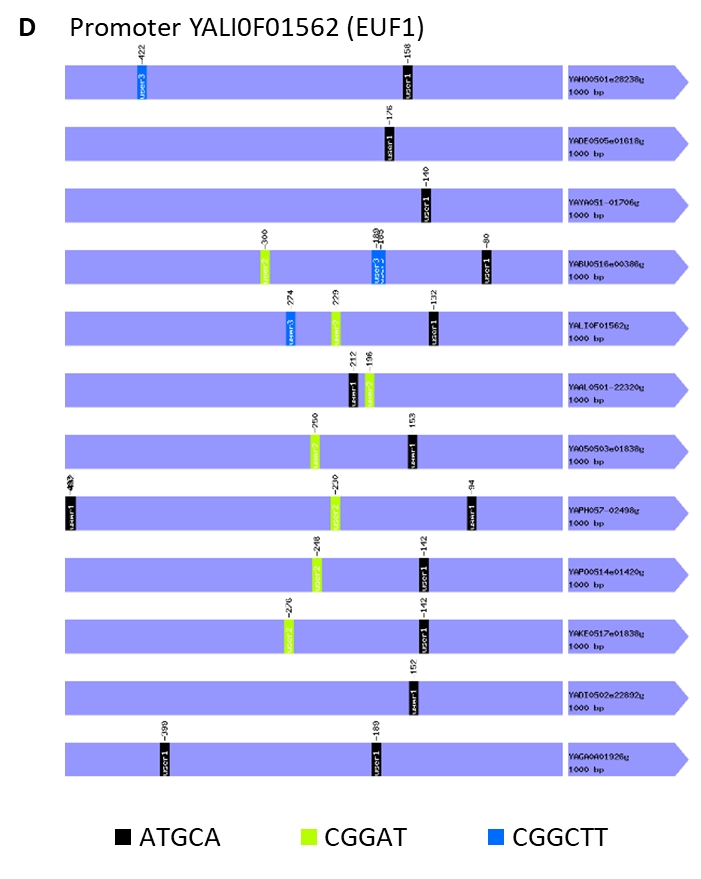


**Figure S2.** Promoter regions of genes from erythritol utilization cluster with the localizations of repeated motifs found by YEASTRACK+.
